# Depression and oral health-related quality of life: A longitudinal study

**DOI:** 10.3389/fpubh.2023.1072115

**Published:** 2023-02-09

**Authors:** Nataliya Nerobkova, Eun-Cheol Park, Sung-In Jang

**Affiliations:** ^1^Department of Public Health, Graduate School, Yonsei University, Seoul, Republic of Korea; ^2^Institute of Health Services Research, Yonsei University, Seoul, Republic of Korea; ^3^Department of Preventive Medicine, Yonsei University College of Medicine, Seoul, Republic of Korea

**Keywords:** CESD, GOHAI, older adults, geriatrics, KLoSA, Korea

## Abstract

**Objectives:**

Geriatric oral health-related quality of life is a relatively new but rapidly growing concept as it is directly related to the general wellbeing and self-esteem of older adults. This study assessed the impact of worsening depression symptoms on oral health-related quality of life using representative nationwide data of Korean older adults.

**Methods:**

This study comprised a longitudinal sample of older adults aged ≥60 from the Korean Longitudinal Study of Aging (2016–2020). After applying the exclusion criteria, 3,286 participants were included in the study. Depression status was determined through the biennial assessment of the short-form Center for Epidemiologic Studies Depression Scale (CESD-10); oral health was measured using the Geriatric Oral Health Assessment Index (GOHAI). We employed the lagged general estimating equations to assess the temporal effect of the CESD-10 score change on the GOHAI score.

**Results:**

A decrease in CESD-10 score over a 2-year period was significantly associated with a decrease in GOHAI score in men and women: β = −1.810 and β = −1.278, respectively (*p*-values < 0.0001). Furthermore, compared to the same or improved CESD-10 score, worsening of the score on 1–2 points detected the β = −1.793 in men and β = −1.356 in women, and worsening on ≥3 points: β = −3.614 in men and β = −2.533 in women.

**Conclusions:**

This study found that depression exacerbation is negatively associated with oral health-related quality of life in later life. Further, a more significant worsening of depression symptoms was correlated with lower scores for oral health-related quality of life in our study population.

## 1. Introduction

The world is progressing toward an aging population owing to the annual increase in average life expectancy. More medical researchers have focused on the burden that comes with an aging population and have defined processes leading to healthier and more successful aging (1). This increased emphasis on the aging population highlights the importance of careful consideration and attention to the health and quality of life of older adults.

Health-related quality of life is a multidimensional construct (2, 3) that includes oral health-related quality of life (4, 5), drawing the attention of researchers to geriatric dentistry (6). Oral disorders significantly affect the wellbeing and life satisfaction of older adults (7). In the age group >60 years, conditions associated with oral health problems are one of the leading causes of general morbidity (8). A decrease in social activity, somatic pathology, and a difficult financial situation impede full-fledged dental care for older people. Appropriate and timely access to oral health care is warranted to assess these problems and improve the overall quality of life.

In particular, South Korea (hereafter, Korea) reported that older individuals with poor oral health are more likely to experience depressive episodes (9). A cross-sectional study by Mun et al. emphasized the impact of depression and oral dryness on oral health-related quality of life (10). Globally, depression is a major public health concern and a leading cause of adverse health outcomes. Individuals with depression experience various symptoms, including reduced physical activity; some patients even show an extreme form of inactivity due to catatonia (11). Hence, researchers and clinicians have made efforts to prevent, reduce, and ameliorate depressive symptoms in populations.

The association between depression and oral health-related quality of life has been addressed in several cross-sectional and longitudinal studies (12); however, little attention has been paid to the time-changing aspect of depression status concerning the geriatric worsening of oral health-related quality of life. Extant literature hypothesizes that comparable biological and behavioral mechanisms are associated with both depression and oral health (13). However, several studies have explicitly examined the bidirectional relationship between depression and oral health and reported controversial results.

Therefore, the impact of the time-changing depressive symptoms on oral health-related quality of life among older Korean adults remains understudied. Moreover, some previous studies present limitations given their cross-sectional design and lack of representative samples. Thus, we hypothesized that individuals with worsening depression over time are more susceptible to experiencing a worse oral health-related quality of life. Furthermore, the greater the exacerbation of depression, the worse the oral health outcomes. Using the short-form Center for Epidemiologic Studies Depression Scale (CESD-10) and the Geriatric Oral Health Assessment Index (GOHAI), we systematically explored the effect of depression status changes on oral health-related quality of life among Korean older adults, based on a nationwide, longitudinal study after adjusting for assumed confounders that might affect the results.

## 2. Methods

### 2.1. Data source and sample

The data examined in the current study was extracted from the Korean Longitudinal Study of Aging (KLoSA), a longitudinal panel survey performed every even-numbered year since 2006. KLoSA provides the data of a representative nationwide sample of older adults over 45 years. The total number of participants surveyed in 2006 was 10,254, with the sample retention rate in 2020 of 63.3%. The main objective of the KLoSA is to provide data that can facilitate the collection of information on the demographic, economic, social, and psychological status and health conditions of middle- and old-aged people and the development of effective policies. In this study, we used data from the last three waves (2016, 2018, and 2020), as GOHAI was first employed in the survey in 2018. The application of exclusion criteria (participants aged < 60 years, missing data of variables, and lack of follow-up) led to the inclusion of 3,286 participants (1,488 men and 1,798 women) in the baseline year.

### 2.2. Depression status

The short-form Center for Epidemiologic Studies Depression Scale (CESD-10) was used to measure depressive symptoms. The CESD-10 is a validated construct used as a screening tool to identify major depressive symptoms in older adults worldwide and in Korea (14, 15). Respondents were asked to answer 10 questions about their condition. Responses were made using a binomial scoring system, coded 0–1 (no/yes), and calculated as a total score of 10 points. Higher scores indicated greater distress and higher severity of depressive symptoms. The continuous variable of depression status change was categorized into two groups as the change of CESD-10 score over time: (1) same or improved and (2) worsened; the analyses were conducted assuming continuous changes in the recorded values within the two groups.

### 2.3. Oral health-related quality of life

The outcome variable, “oral health-related quality of life,” was identified using the Geriatric Oral Health Assessment Index (GOHAI), which has been introduced for general use to measure the quality of life related to oral health and has proven its validity and reliability in adult samples worldwide, including Korea (16) and other Asian countries (17, 18). The GOHAI is based on responses to a 12-item self-administered questionnaire measured on a 6-point scale (0–5 points), while three positive questions are calculated by reversing the scores. A higher GOHAI score indicates a higher quality of life related to positive oral health, with the highest possible score of 60 points. To analyze the relationship between the change in depressive symptoms and the GOHAI score, changes in both scales over the previous year were recorded.

### 2.4. Covariates

We added data on sociodemographic characteristics and health-related factors as potential covariates in this study. Sociodemographic characteristics included gender (men and women), age (60–64, 65–69, 70–74, and ≥75 years), educational level (middle school or below and high school or above), marital status (married and not married), and income level per month in quartiles (low, middle-low, middle-high, and high). Additionally, we considered the participants' regions of residence (urban and rural areas). Health-related factors included the number of chronic medical conditions, smoking status (non-smoker and smoker), handgrip strength (normal and abnormal), cognitive function (normal and abnormal), and body mass index (BMI). KLoSA provides data on comorbidities, including hypertension, diabetes mellitus, cancer, lung disease, heart disease, and cerebrovascular disease. We grouped them into three categories depending on the number of diseases a participant had (none, 1, or ≥2 diseases). Handgrip strength was measured in kilograms considering sex-specific thresholds (<24 kg for men and <15 kg for women). Cognitive function was assessed through the Mini-Mental State Examination (MMSE) score; with a total score of 30, the MMSE's cut-off level for cognitive impairment is below 24. Furthermore, we considered participants' life satisfaction (bad, normal, and good).

### 2.5. Statistical analysis

Lagged generalized estimating equation (GEE) analyses were performed and controlled for covariates to provide estimates for the GOHAI according to the 2-year change in CESD-10 scores. To examine gender-specific differences, all statistical analyses were performed separately for men and women. The GEE model allows for repeated measurement analysis of longitudinal panel survey data and considers the correlation within the subject to generate regression coefficients (β), standard errors (S.E.), and the corresponding *p*-value.

Subgroup analyses were performed to determine the relationship between the CESD-10 and GOHAI. We estimated the lagged GEE analyses for depression transitions for each GOHAI question. Furthermore, we performed a sensitivity analysis to determine the relationship between specific changes in the CESD-10 score (worsening on 1–2 points and ≥3 points) and the GOHAI. All differences were considered statistically significant with *p*-values of <0.05. Data analyses were performed using the SAS 9.4 software (SAS Institute Inc., Cary, NC, USA).

## 3. Results

The sex-stratified baseline characteristics of the study population are presented in Table 1. A total of 3,286 individuals were included in the survey during the baseline year (1,488 men and 1,798 women). In the unadjusted analysis, the mean difference in depression status groups and GOHAI scores was ~3 points for men and women.

**Table 1:** Baseline characteristics of the study population according to Geriatric Oral Health Assessment Index (2016 → 2018).

**Variables**	**Geriatric Oral Health Assessment Index**									
	**Men**				**Women**					
	***N***	**%**	**Mean** ±**S.D**.		***P*-value**	***N***	**%**	**Mean** ±**S.D**.		***P*-value**
**Total (*N* = 3,286)**	**1,488**	**100.0**	**38.61**	**8.46**		**1,798**	**100.0**	**38.27**	**8.19**	
**Change in CESD-10 score**					< 0.0001					< 0.0001
Same or improved	1,055	70.9	39.61	8.68		1,203	66.9	39.37	8.28	
Worsened	433	29.1	36.19	7.38		595	33.1	36.04	7.55	
**Age**					0.0097					< 0.0001
60–64	285	19.2	41.239	8.54		393	21.9	41.489	7.55	
65–69	359	24.1	40.326	8.03		420	23.4	39.612	7.61	
70–74	311	20.9	38.344	7.71		370	20.6	38.165	7.66	
≥75	533	35.8	36.214	8.46		615	34.2	35.354	8.31	
**Region**					0.1531					0.2669
Urban area	611	41.1	39.321	8.25		734	40.8	39.04	7.86	
Rural area	877	58.9	38.121	8.58		1,064	59.2	37.736	8.38	
**Educational level**					0.0013					< 0.0001
Middle school or below	440	29.6	36.018	8.76		1,036	57.6	36.506	8.30	
High school or above	1,048	70.4	39.703	8.09		762	42.4	40.664	7.41	
**Employment status**					0.2128					0.5645
Employed	636	42.7	40.097	7.94		420	23.4	39.631	8.13	
Non-employed	852	57.3	37.506	8.67		1,378	76.6	37.853	8.17	
**Marital status**					0.8013					0.7178
Married	1,352	90.9	38.811	8.47		1,123	62.5	39.251	7.77	
Not married	136	9.1	36.654	8.10		675	37.5	36.633	8.61	
**Household income per month**					0.0450					0.5128
Quartile 1 (low)	387	437.0	36.167	9.06		742	41.3	36.636	8.64	
Quartile 2	412	390.0	38.287	7.78		426	23.7	38.761	7.33	
Quartile 3	411	391.0	40.302	8.36		381	21.2	39.793	7.49	
Quartile 4 (high)	333	270.0	40.6	7.47		249	13.8	39.956	8.40	
**Chronic disease**					0.1459					0.6289
None	553	37.2	39.687	8.03		637	35.4	39.143	7.99	
1	521	35.0	38.668	8.47		688	38.3	38.222	8.01	
≥2	414	27.8	37.111	8.80		473	26.3	37.156	8.59	
**Smoking status**					0.0013					0.0612
Non-smoker	471	31.7	39.584	8.32		1,737	96.6	38.366	8.13	
Smoker	1,017	68.3	38.164	8.50		61	3.4	35.475	9.43	
**Handgrip strength**					0.0056					0.0023
Normal	1,196	80.4	39.334	8.25		1,485	82.6	39.024	7.93	
Abnormal	292	19.6	35.664	8.68		343	19.1	34.708	8.50	
**Cognitive function**					< 0.0001					< 0.0001
Normal	1,156	77.7	39.843	8.12		1,213	67.5	40.001	7.65	
Abnormal	332	22.3	34.331	8.23		585	32.5	34.675	8.13	
**BMI**					0.7552					0.7872
Normal	1,429	96.0	38.728	8.38		1,713	95.3	38.382	8.18	
Abnormal	59	4.0	35.831	9.87		85	4.7	35.965	8.22	
**Satisfaction of life**					0.0002					< 0.0001
Bad	157	10.6	34.459	8.21		261	14.5	34.375	8.76	< 0.0001
Normal	970	65.2	38.354	8.27		1,143	63.6	38.183	7.83	
Good	361	24.3	41.119	8.27		394	21.9	41.094	7.76	

Table 2 presents the findings of the lagged GEE model analysis of the association between changes in CESD-10 and GOHAI scores. We noted that, in both men and women, compared to the same or improved depression status, worsened depression showed regression coefficients of −1.810 in men and −1.278 in women, which were highly significant at *p*-values < 0.0001. A decrease in CESD-10 score was significantly associated with a decrease in GOHAI score in both genders.

**Table 2:** Generalized linear model using the generalized estimating equations with Geriatric Oral Health Assessment Index in 2018.

**Variables**	**Geriatric Oral Health Assessment Index**					
	**Men**			**Women**		
	**β**	**S.E**.	***P*-value**	**β**	**S.E**.	***P*-value**
**Change in CESD-10 score**						
Same or improved	ref.			ref.		
Worsened	−1.810	0.3018	< 0.0001	−1.278	0.2577	< 0.0001

The findings of the independent subgroup analysis of the variables associated with the effect of changes in depression symptoms and GOHAI scores are presented in Table 3. The results indicated that men with worsened depressive symptoms aged ≥75 years and women in the 70–74 age group showed statistically significant regression coefficients: β = −1.862 in men and β = −1.790 in women, respectively. Furthermore, low handgrip strength was strongly associated with lower GOHAI scores in the group with worsened depressive symptoms: β = −2.009 in men and β = −2.468 in women.

**Table 3:** Subgroup analysis using the GEE of Geriatric Oral Health Assessment Index with depression status in 2020.

**Variables**	**Geriatric Oral Health Assessment Index**							
	**Men**				**Women**			
	**Change in CESD-10 score**							
	**Same or improved**	**Worsened**			**Same or improved**	**Worsened**		
	**β**	**β**	**S.E**.	***P*-value**	**β**	**β**	**S.E**.	***P*-value**
**Age**								
60–64	Ref.	−2.008	1.0770	0.062	Ref.	−0.872	0.7237	0.228
65–69	Ref.	−0.395	0.8357	0.637	Ref.	−1.462	0.8568	0.088
70–74	Ref.	−0.133	0.8945	0.882	Ref.	−1.790	0.7159	0.012
≥75	Ref.	−1.862	0.7840	0.018	Ref.	−0.831	0.6293	0.187
**Region**								
Urban area	Ref.	−2.397	0.7124	0.001	Ref.	−1.790	0.6185	0.004
Rural area	Ref.	−0.034	0.5762	0.953	Ref.	−0.999	0.4563	0.029
**Employment status**								
Employed	Ref.	0.010	0.7164	0.989	Ref.	−1.020	0.8673	0.239
Non-employed	Ref.	−1.709	0.5812	0.003	Ref.	−1.213	0.4099	0.003
**Handgrip strength**								
Normal	Ref.	−0.540	0.5060	0.286	Ref.	−0.745	0.4003	0.063
Abnormal	Ref.	−2.009	0.9684	0.038	Ref.	−2.648	0.8660	0.002

Table 4 shows the lagged GEE model analysis results for the effect of a decreased CESD-10 score on each GOHAI question. The question related to the limitation of contact with other people due to teeth or dentures showed the most significant association with worsening depression: β = −0.270 in men and β = −0.319 in women. However, little to no discomfort while eating was associated with an improved depression status (β = 0.367 in men and β = 0.328 in women).

**Table 4:** Subgroup analysis using the GEE of Geriatric Oral Health Assessment Index questions with depression status.

**Variables**	**Men**				**Women**			
	**Change in CESD-10 score**							
	**Same or improved**	**Worsened**			**Same or improved**	**Worsened**		
	**β**	**β**	**S.E**.	***P*-value**	**β**	**β**	**S.E**.	***P*-value**
**Geriatric Oral Health Assessment Index (questions)**								
1. How often did you limit the kinds or amounts of food you eat because of problems with your teeth or dentures?	Ref.	−0.238	0.0664	0.0003	Ref.	−0.289	0.0518	< 0.0001
2. How often did you have trouble biting or chewing different kinds of food, such as firm meat or apples?	Ref.	−0.221	0.0635	0.0005	Ref.	−0.237	0.0550	< 0.0001
3. How often were you able to swallow comfortably?^*^	Ref.	0.279	0.0854	0.0011	Ref.	0.317	0.0691	< 0.0001
4. How often have your teeth or dentures prevented you from speaking the way you want?	Ref.	−0.221	0.0684	0.0013	Ref.	−0.232	0.0555	< 0.0001
5. How often were you able to eat anything without feeling discomfort?^*^	Ref.	0.367	0.0836	< 0.0001	Ref.	0.328	0.0690	< 0.0001
6. How often did you limit contact with other people because of the condition of your teeth or dentures?	Ref.	−0.270	0.0566	0.0006	Ref.	−0.319	0.0566	< 0.0001
7. How often were you pleased or happy with the looks of your teeth and gums or dentures?^*^	Ref.	0.007	0.0818	0.9292	Ref.	−0.018	0.0677	0.7869
8. How often did you use medication to relieve pain or discomfort around your mouth?	Ref.	−0.202	0.0671	0.0027	Ref.	−0.227	0.0545	< 0.0001
9. How often were you worried or concerned about problems with your teeth, gums, or dentures?	Ref.	−0.117	0.0644	0.0698	Ref.	−0.218	0.0559	< 0.0001
10. How often did you feel nervous or self- conscious because of problems with teeth, gums, or dentures?	Ref.	−0.095	0.0658	0.1493	Ref.	−0.189	0.0555	0.0007
11. How often did you feel uncomfortable eating in front of other people because of problems with your teeth or dentures?	Ref.	−0.176	0.0641	0.0060	Ref.	−0.203	0.0537	0.0002
12. How often were your teeth or gums sensitive to hot, cold, or sweet foods?	Ref.	−0.161	0.0657	0.0143	Ref.	−0.226	0.0524	< 0.0001

* Three positive questions were calculated by reversing the scores in main analysis.

The sensitivity analysis results for the 2-year lagged transitions in terms of CESD-10 score changes on the GOHAI score are presented in Table 5. Compared to the same or improved CESD-10 score, worsening of the score by 1–2 points detected regression coefficients of −1.793 in men and −1.356 in women, and worsening by ≥3 points −3.614 in men and −2.533 in women, respectively.

**Table 5:** Generalized linear model using the generalized estimating equations with Geriatric Oral Health Assessment Index and the short form of Center for Epidemiological Studies Depression-10 scores change.

**Variables**	**Geriatric Oral Health Assessment Index**					
	**Men**			**Women**		
	**β**	**S.E**.	***P*-value**	**β**	**S.E**.	***P*-value**
**Change in CESD-10 score**						
Same or improved	Ref.			Ref.		
Worsened on 1–2 points^*^	−1.793	0.3146	< 0.0001	−1.356	0.2665	< 0.0001
Worsened on ≥3 points^**^	−3.614	0.6414	< 0.0001	−2.533	0.4950	< 0.0001

* Mild worsening.

** Severe worsening of depressive symptoms.

## 4. Discussion

The substantial increase in the average life expectancy of people worldwide poses a challenge to public, social, and behavioral sciences, and medicine. Research has identified risk factors for poor oral health-related quality of life in older adults (19, 20). Strategies and interventions developed through a precise examination of the above-mentioned factors are expressly warranted to ensure that a greater proportion of our population ages without oral health-related discomfort.

The results contribute to the evidence that depression exacerbation during the previous 2 years is significantly associated with poor oral health-related quality of life. We found that the participants whose CESD-10 scores decreased had reported lower GOHAI scores than those whose depression status had remained the same or improved in the same period (Table 2). Furthermore, we found that the severity of depression exacerbation was negatively correlated with GOHAI scores in our study population. Those with a decrease in the CESD-10 scores of ≥3 points reported a greater decrease in overall geriatric oral health-related quality of life than those with a decrease of 1–2 points or with the same or improved depression condition (Table 5).

Our results are consistent with those of previous studies, namely, that depression was associated with a worsening of overall oral health-related quality of life. For example, one study found that the oral health impact profile was significantly associated with depression, stress, and self-esteem in community-dwelling middle-aged women (21). Although this was a cross-sectional study, the Pearson correlation coefficient of the correlation with depression (*r* = −0.560, *p* < 0.001) revealed a highly statistically significant outcome. Furthermore, another cross-sectional study involving participants with suspected dementia in Korea found that oral dryness and depression among older adults influenced oral health-related quality of life (10). A cross-sectional study using longitudinal data conducted in Germany by Hassel et al. reported that oral health-related quality of life was significantly linked to wellbeing and depression in later life, assuming that subjective health could mediate this relationship (22).

In the present study, a subgroup analysis of independent variables indicated that weak handgrip strength and worsened depression were related to lower oral health-related quality of life (Table 3). Previous research has observed an association between the GOHAI, its pain and discomfort dimension, and handgrip strength, suggesting that oral health problems associated with discomfort may be significant indicators of a decline in body muscle strength (23). The dependent subgroup analysis on the domains of the GOHAI revealed the link between the worsening of depression and limitation of social contacts due to oral problems, which supports the results of a cross-sectional study in Japan that assumed that depressive symptoms were significantly associated with social engagement, with greater associations in less mentally frail populations (24).

The etiology of the association between depression and oral health-related quality of life has not yet been well-elucidated. However, several possible biological (or bacterial and immunological) and behavioral mechanisms have been proposed. Changes in salivary immunity have been documented to be negatively associated with stressful life events (25). Therefore, a high life stress load would seem to compromise either production or transport of the saliva secretion rate and given the prominent role of saliva in immune defense on mucosal surfaces (26), it may contribute to the onset of oral health problems. In addition, a positive association between the growth of lactobacilli and the use of anti-depressant medication when dental caries was considered an oral health outcome has been found (27, 28). The association between high lactobacillus counts and depressive symptoms suggests that depressed individuals are at a higher risk of developing caries and other dental diseases. The behavioral components were suggested to be related to the positive contribution of depression to poor oral health by acquiring and maintaining harmful oral habits (29, 30) and poor dental health (31).

The present study has several strengths and limitations. The main limitation of our study was that most of the data were self-reported and collected through surveys. Although we employed globally used and validated indexes for our main variables, we cannot exclude the risk of biased results. We also attempted to minimize the potential bias attributable to health selection by controlling for baseline health status. Second, the data of those who did not answer the essential covariate questions were excluded, which might have led to an underestimation of the conditions of some participants. Third, biological risk factors that might significantly affect the adjustment of variables may have been overlooked. Finally, causation could not be established due to the lack of a prospective design. We used the change in the depression condition between the two waves and analyzed its association with oral health-related quality of life in the subsequent wave to minimize the risk of reciprocal causation. However, the lack of data on GOHAI in previous years and poor oral-health-related quality of life might affect the severity of depression. Thus, future studies using a prospective design are needed to establish the causal relationship between changes in depressive symptoms and oral health-related quality of life.

Nonetheless, the strengths of our study include its longitudinal design, with the results representing the Korean older adult population of over 60 years. These findings can be applied to the general Korean population to establish healthcare policies or conduct future studies. Second, we used standardized tools to measure depression and oral health-related quality of life, which provides a substantial basis for future research. Finally, we used the change in depression rather than a timepoint depression estimation; thus, the results on depression as a changing over-time variable add novel information to the existing body of literature.

## 5. Conclusion

In conclusion, this study was conducted to assess the impact of changing depression conditions over time on geriatric oral health-related quality of life using longitudinal nationwide data of older adults in Korea. The findings of this study suggest that depression is negatively associated with oral health-related quality of life in later life. Participants with worsened depressive symptomology were more susceptible to poor oral health-related quality of life than those whose depression status remained the same or improved during the same period. The results also demonstrated that the severity of depression exacerbation was negatively correlated with GOHAI scores in our study population. Providing resources to assist older people with depression and on-time access to oral care may help prevent psychological distress and improve their chances of experiencing an improved oral health-related quality of life. Future studies addressing the underlying mechanisms of this association as well as the bidirectional preventive effects may provide valuable tactics for treating and preventing poor oral health-related quality of life.

## Data availability statement

The datasets used during the study are publicly available in the Korean Longitudinal Study of Aging Repository via https://survey.keis.or.kr/klosa/klosa01.jsp. Any queries should be directed to the corresponding author.

## Ethics statement

The KLoSA survey was approved by the National Statistical Office and Institutional Review Board of the Korea Centers for Disease Control and Prevention, with all methods following the relevant guidelines and regulations. As the KLoSA database is open for public use, ethical approval was not required for the study. All participants provided written informed consent to participate in the KLoSA survey and agreed to be used in further scientific research. The data were anonymized and de-recognizable with no personal information, with cautious protection on confidentiality. The patients/participants provided their written informed consent to participate in this study.

## Author contributions

NN and S-IJ: conceptualization and visualization. NN, E-CP, and S-IJ: data curation and writing—review and editing. NN: formal analysis and writing—original draft. NN and E-CP: methodology. All authors contributed to the article and approved the submitted version.
